# An integrative quantifier of multistability in complex systems based on ecological resilience

**DOI:** 10.1038/srep16196

**Published:** 2015-11-05

**Authors:** Chiranjit Mitra, Jürgen Kurths, Reik V. Donner

**Affiliations:** 1 Potsdam Institute for Climate Impact Research, Transdisciplinary Concepts & Methods - Research Domain 4, Potsdam, 14412, Germany; 2 Humboldt University of Berlin, Department of Physics, Berlin, 12489, Germany; 3 University of Aberdeen, Institute for Complex Systems and Mathematical Biology, Aberdeen, AB24 3UE, United Kingdom; 4 Nizhny Novgorod State University, Department of Control Theory, Nizhny Novgorod, 606950, Russia

## Abstract

The abundance of multistable dynamical systems calls for an appropriate quantification of the respective stability of the (stable) states of such systems. Motivated by the concept of ecological resilience, we propose a novel and pragmatic measure called ‘integral stability’ which integrates different aspects commonly addressed separately by existing local and global stability concepts. We demonstrate the potential of integral stability by using exemplary multistable dynamical systems such as the damped driven pendulum, a model of Amazonian rainforest as a known climate tipping element and the Daisyworld model. A crucial feature of integral stability lies in its potential of arresting a gradual loss of the stability of a system when approaching a tipping point, thus providing a potential early-warning signal sufficiently prior to a qualitative change of the system’s dynamics.

The ubiquity of dynamical systems exhibiting multistability can hardly be further exaggerated. The human brain^1^, ecosystems^2^, ice sheets^3^, optical ring cavities^4^, time-delay systems^5^, synthetic genetic networks^6^, chemical oscillators^7^, etc. constitute notable examples among a large body of multistable systems^8^.

Linear stability analysis, based on the ‘local’ assessment of the sign and magnitude of the Lyapunov exponents in the attractor’s neighbourhood, in conjugation with the recently proposed ‘global’ measure of basin stability (BS), quantified as the volume of the basin of attraction^9^, constitute state of the art methods of gauging stability in dynamical systems theory. In this work, we identify various aspects characterizing the stability of a multistable dynamical system and demonstrate that linear stability and BS are unable to capture all of them. Subsequently, we propose the novel and pragmatic measure of ‘integral stability’ (IS) for holistically inferring stability, overcoming the main insufficiencies of the existing metrics.

The foundation of IS rests upon the concept of ‘resilience’, introduced into the ecological literature by C. S. Holling in 1973^10^. There are at least two different definitions of resilience of a system, depending on the assumption of the presence of one or multiple stable states in the system. We briefly review these two definitions of resilience, prior to utilization of the concept. The first definition due to Pimm^11^ termed *engineering resilience* by Holling^12^, focussing on stability near an equilibrium state, is measured using the resistance to disturbance and speed of return to its equilibrium, following a perturbation. The second definition due to Holling^12^ termed *ecological resilience*, is defined as the capacity of a system to absorb disturbance and reorganize while undergoing change so as to still retain essentially the same function, structure, identity and feedbacks^13^. It emphasizes upon conditions far from any steady state, where instabilities can flip a system into another regime, i.e., to another stability domain^12^. In this case, the resilience of a system is measured by its capacity to remain in the same basin of attraction in the face of perturbations. The two definitions of resilience reflect the different aspects of stability being emphasized. Engineering resilience implicitly assumes global stability, i.e., the existence of only one equilibrium state, or, if other operating states exist, they should be avoided by applying safe guards^14^. On the other hand, ecological resilience presumes the existence of multiple stable states and the tolerance of the system to disturbances that facilitate transitions among the stable states. Given our interest in quantifying the stability of multistable dynamical systems, henceforth, we primarily focus and build upon ecological resilience.

Walker *et al.*^13^ identifies ‘latitude’ (*L*), ‘resistance’ (*R*) and ‘precariousness’ (*Pr*) as the three crucial aspects of ecological resilience. Below, we turn to the definitions of *L*, *R* and *Pr* proposed by Walker *et al.*^13^ and redefine them in a rigorous dynamical systems context for usage throughout this paper as a basis for the formal definition of IS:

- *Latitude L* describes “the maximum amount the system can be changed before losing its ability to recover; basically the width of the basin of attraction”^13^. Here, we consider the volume of the basin of attraction (i.e., BS) as a quantitative measure of the latitude of a stable state.
- *Resistance R* refers to “the ease or difficulty of changing the system; related to the topology of the basin - deep basins of attraction indicate that greater forces or perturbations are required to change the current state of the system away from the attractor”^13^. Pimm defined resistance as “the degree to which a variable is changed following a perturbation”, and resistant systems as ones which “change less under a given disturbance”^11^. Qualitatively, we identify the resistance of a system with its capacity of overcoming changes, following a perturbation.
- More precisely, each point in the state space of a deterministic stationary multistable dynamical system is associated with a unique trajectory approaching a particular attractor. A perturbation drives the system to a different point in the state space, associated with a different trajectory. Building upon our qualitative definition of resistance, and combining it with engineering resilience, we here define resistance at a particular point in the state space as the instantaneous rate at which the system converges to the unperturbed trajectory following a perturbation. Subsequently, we associate the resistance at a particular point in the state space with the local Lyapunov exponents evaluated at the respective point in state space^15^. For any point, the negative of the local Lyapunov exponents measure the rate of convergence of nearby trajectories to the trajectory starting at the state space point in question. Further, the growth of separation in the direction corresponding to the largest among the local Lyapunov exponents overwhelms the growth of separation along other directions. Subsequently, we quantify the resistance at any point in state space as the negative of the largest local Lyapunov exponent, evaluated at the respective point. The detailed procedure for calculating the local Lyapunov exponents is described in the Methods section.
- *Precariousness Pr* addresses “the current trajectory of the system, and how close it currently is to a limit or threshold which, if breached, makes recovery difficult or impossible”^13^. In this paper, we consider the precariousness of a stable state as being the minimum perturbation required to drive the system residing on the attractor corresponding to the stable state outside its basin of attraction. This definition of the precariousness of a stable state corresponds to the recently proposed definition of ‘stability threshold’ by Klinshov *et al.*^16^. We direct the reader to the algorithm proposed by Klinshov *et al.* for calculating this stability threshold.

The exemplary stability landscape (comprising the basins of attraction of all stable states of a system and the boundaries separating them) in Fig. 1 serves as a heuristic device for illustrating the original conceptualizations of *L*, *R* and *Pr* as proposed by Walker *et al.*^13^ for a basin of attraction of a dynamical system consisting of two state variables. We refer the reader to the review by Beisner *et al.*^17^ for further understanding of the metaphor of stability landscapes as used in ecology. We emphasize that the redefinitions of *L*, *R* and *Pr* constitute the crucial aspects characterizing the stability of attractors of a multistable dynamical system.

Linear stability analysis provides a (locally restrictive) measure of resistance in the immediate neighbourhood of the attractor and does not account for resistance at the global level. On the other hand, BS, an exclusively latitude-based measure, does not consider resistance as well as the position of the attractor within the basin, i.e., precariousness (illustrated with the example of an Amazonian vegetation model in the Results section). For example, for a system with the same basin size for two different parameter values, BS is the same irrespective of the dynamics (leading to possibly different resistances). As another example, consider a system with the same basin size for two different parameter values. In one case, the attractor is at the centre of the basin, thus making it equally vulnerable to perturbations in all directions implying relatively high precariousness. In the other case, assume that the attractor is very close to the basin boundary, thus making it highly vulnerable to perturbations in the direction of closest proximity to the basin boundary implying relatively low precariousness. Clearly, BS is unable to identify this change in the vulnerability of the system to perturbations for the two different parameter values. Thus, the existing methods of linear stability and BS do not individually and collectively capture all three crucial aspects of multistability, motivating the introduction of a new generally applicable measure integrating *L*, *R* and *Pr*.

BS has been identified as a potential tool for assessing multistable climate tipping elements as demonstrated by its application to an Amazonian vegetation model^9^. Although BS captures the qualitative change in the system’s dynamics in terms of a sudden decline in its value when approaching the tipping point, it does not provide a convincing early-warning signal prior to the change. On the other hand, IS exhibits a gradual loss in its value when approaching the tipping point, thus providing a potential early-warning signal prior to such critical transitions. This feature of IS (demonstrated with examples in the subsequent sections) turns out to be one of the key advantages of the measure.

## Integral Stability Measure

Consider an *N*-dimensional (continuous or discrete) dynamical system represented by the state vector **x**(*t*) = (*x*_1_, *x*_2_, …, *x*_*N*_)^T^ exhibiting *M* stable attractors 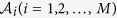. The precariousness 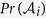 of the *i*^*th*^ attractor is the minimum perturbation required to drive the system presently residing on the attractor 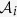, outside its basin of attraction 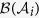. Thus,


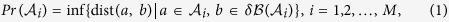


where dist(⋅, ⋅) is the Euclidean distance and 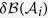 is the border of the basin of attraction of the attractor 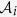
16.

Let *λ*(**x**) = {*λ*_1_, *λ*_2_, …, *λ*_*N*_} be the set of *N* local Lyapunov exponents evaluated at the state **x** (see the Methods section). The resistance *R*(**x**) at the state **x** is the negative of the largest local Lyapunov exponent of the trajectory starting at **x**. Thus,


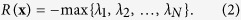


We now define a measure of *integral stability* 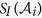 of the *i*^th^ attractor as,


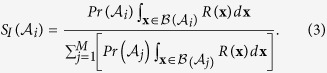


The integral over the basin of attraction of the *i*^th^ attractor with 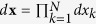 as the differential volume element keeps track of the latitude.

By defining IS in the particular form of equation (3), we assume that the ingredients of *L*, *R* and *Pr* carry equal weights in quantifying the stability of any particular attractor. Subsequently, we multiply the ingredients together and integrate them over the entire basin of the attractor under investigation. Moreover, normalizing the product facilitates the quantitative comparison of the respective stability of an attractor (between 0 and 1) when modulating a particular parameter of the system. The exact value of the product of *L*, *R* and *Pr* is no longer important but only the value of IS of the attractor under question compared to that of the other attractors. Finally, normalization ensures that IS is a dimensionless ratio, which nullifies the effect of units of measurement to some extent.

## Results

In the following, we demonstrate the potential of the proposed measure of IS in quantifying the stability of the attractors of exemplary multistable dynamical systems.

### Damped Driven Pendulum

We consider the stability of a classical damped pendulum driven by a constant angular acceleration. The dynamical equations of the system read


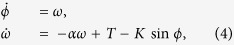


where *ϕ* is the angular position, *ω* is the angular velocity, *α* > 0 is the dissipation coefficient, *T* is the constant angular acceleration and 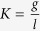, with *g* and *l* being the gravitational acceleration and the length of the pendulum, respectively. For 0 ≤ *T* ≤ *K*, the pendulum has two equilibrium points, *x*_*i*_ = (*ϕ*_*i*_, *ω*_*i*_) for *i* = 1, 2,


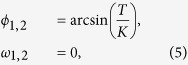


where *ϕ*_1_ is the solution of the arcsin inside 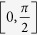 and *ϕ*_2_ = *π* − *ϕ*_1_.

For 0 < *T* < *K* with fixed *K* and *α*, *x*_1_ is a stable equilibrium and *x*_2_ is an unstable saddle. In addition, for *T*_mult_ < *T* < *K*, the pendulum can also converge to a limit cycle, thus exhibiting multistability. On varying *T* with *α* = 0.1 and *K* = 1, we observe the limit cycle becoming stable at *T*_mult_ ≈ 0.13.

**Figure 2:**
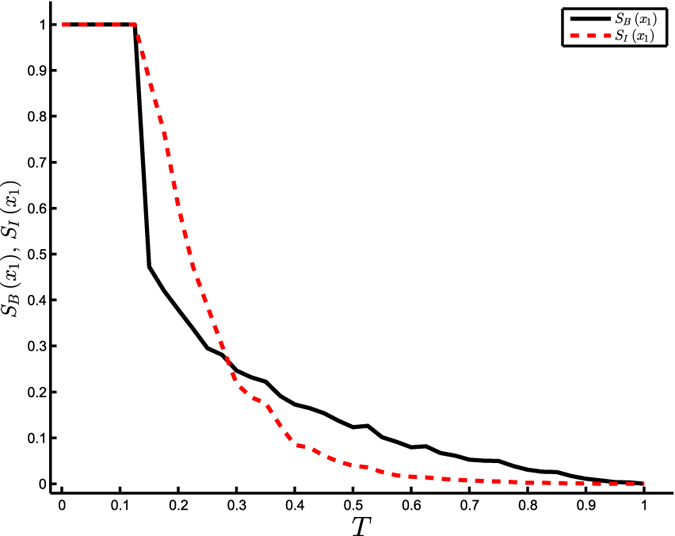
Stability of the damped driven pendulum (equation (4)). Integral stability *S*_*I*_(*x*_1_) and basin stability *S*_*B*_(*x*_1_) of the equilibrium point *x*_1_ vs. *T*, at *α* = 0.1 and *K* = 1.

As already stated in Menck *et al.*^9^, linear stability (i.e., the maximum Lyapunov exponent of the stable equilibrium *x*_1_) is not a reliable proxy for detecting the pendulum’s transition between *x*_1_ and the limit cycle under large perturbations (Supplementary Fig. S3 of Menck *et al.*^9^). On the contrary, the measure of IS proposed in equation (3) clearly detects the transition (and closely follows the measure of BS) as evident from Fig. 2.

### Amazonian Vegetation Model

In a second example, we explore the stability of a simple paradigmatic model of an important potential climate tipping element, the Amazonian rainforest^9^,^18^,^19^. The dynamical equations of the model read


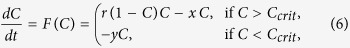


where *C* is the relative forest cover that grows at a rate *r* if *C* > *C*_*crit*_ and dies with a rate *x* (*y*) if *C* > *C*_*crit*_ (*C* < *C*_*crit*_), assuming *r* > *x* (*y*) > 0. *C*_*crit*_ is the critical forest cover threshold, which is assumed to increase linearly with aridity *A*, i.e., *C*_*crit*_ = *C*_1_*A* + *C*_2_, where *C*_1_ and *C*_2_ are arbitrary constants. The aridity is an indicator of the degree of dryness of the climate at a given location. In the following, we set *C*_1_ = 1 and *C*_2_ = 0 for convenience, which implies that *C*_*crit*_ = *A*. Thus, the critical forest cover threshold is set equal to the aridity. This model has two equilibria, the forest state 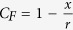 and the savanna state *C*_*S*_ = 0. The equilibrium *C*_*F*_ (*C*_*S*_) exists and is stable if *C*_*F*_ > *C*_*crit*_ (*C*_*crit*_ > 0).

**Figure 3:**
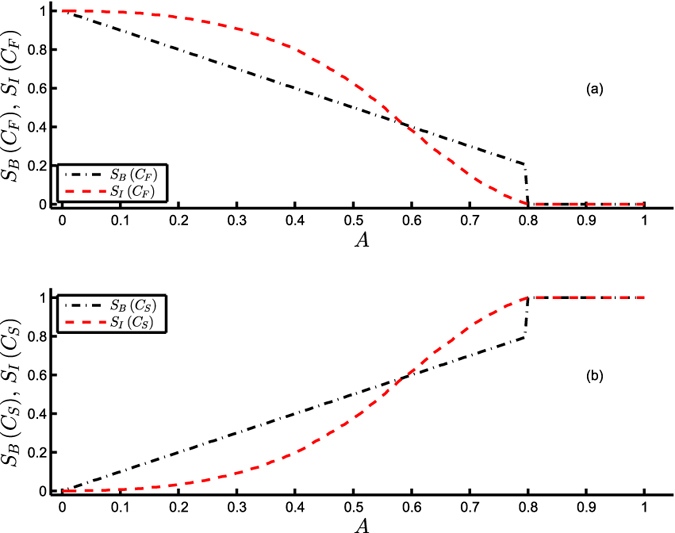
Stability of the Amazonian vegetation model (equation (6)). Integral stability *S*_*I*_ and basin stability *S*_*B*_ of (**a**) the forest state 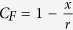 vs. aridity *A* and (**b**) the savanna state *C*_*S*_ = 0 vs. *A*, at *C*_*crit*_ = *A*, *x* = 0.2, *r* = 1.0, *y* = 1.0.

Menck *et al.*^9^ argues that owing to the local nature of linear stability and small-perturbation convergence rate, these measures fail to identify the decrease (increase) in the global stability of the forest (savanna) state *C*_*F*_ (*C*_*S*_) on account of the shrinkage (expansion) of its basin of attraction on increasing the aridity *A*, which is however successfully quantified by BS (Fig. 3). Likewise, IS proposed in equation (3) clearly detects the change in the stability of the forest (savanna) state *C*_*F*_ (*C*_*S*_) as evident from Fig. 3(a)(Fig. 3(b)).

**Figure 4:**
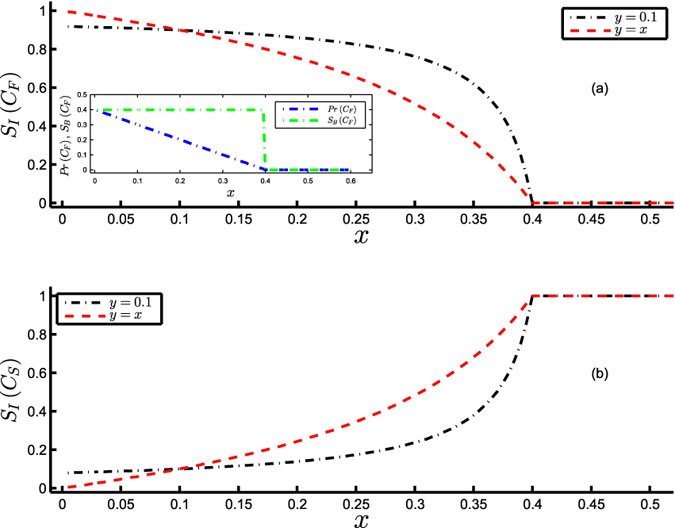
Capturing precariousness and resistance in quantifying the stability of the Amazonian vegetation model (equation (6)). Integral stability *S*_*I*_ of (**a**) the forest state *C*_*F*_ vs. *x* for *y* = 0.1 (black) and *y* = *x* (red) and (**b**) the savanna state *C*_*S*_ vs. *x* for *y* = 0.1 (black) and *y* = *x* (red), at *C*_*crit*_ = *A* = 0.6, *r* = 1.0. The inset in (**a**) illustrates the decreasing value of the precariousness *Pr* (blue) and the constant value of basin stability *S*_*B*_ (green) of the forest state with increasing *x*.

As pointed out in the Introduction, BS is unable to identify the change in the stability of an equilibrium state of a system on varying its position with respect to the basin boundary. For example, the forest state at 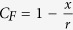 comes closer to the basin boundary at *C*_*crit*_ on increasing *x*, which increases the vulnerability of the forest state to perturbations in the direction of the basin boundary at *C*_*crit*_. The inset in Fig. 4(a) clearly illustrates the decreasing value of the precariousness *Pr*(*C*_*F*_) of the forest state upon increasing *x*, which finally goes to 0 at the point of critical transition. However, it is easy to identify from the inset that the constant value of BS, *S*_*B*_(*C*_*F*_), does not capture this decrease in the precariousness of *C*_*F*_ prior to the transition. This is aptly captured by the proposed measure as evident from the decreasing value of IS of the forest state with rising *x* in Fig. 4(a). In the latter case, the fixed value of *y* implies that the local Lyapunov exponent of the savanna state *C*_*S*_ = 0 is constant and equal to *y*(= 0.1) even as *x* is varied. The above example clearly magnifies the advantage of IS in comparison with linear stability and BS by demonstrating its ability to capture the gradual loss in the stability of an equilibrium state on account of reduced precariousness.

Further, as mentioned in the Introduction, BS does not consider the local dynamics in quantifying the stability of an attractor. For example, it does not quantify the change in the stability of the savanna state *C*_*S*_ = 0 upon varying the value of *y* as this varies the resistance in the neighbourhood of the savanna state. Such changes in the resistance affect the rate of convergence to the equilibrium (identified as ‘engineering resilience’ of the equilibrium state) on being perturbed. Consider the situation when *C*_*crit*_ = *A* = 0.6, *r* = 1.0 and *y* = *x* such that the local Lyapunov exponent of the savanna state is equal to −*y* = −*x*, implying an increase in the resistance (in this case, increase in the rate of convergence) in the neighbourhood of *C*_*S*_ with increasing *x*. Subsequently, at any value of *x*, the situation when *y* = *x* leads to larger stability of the savanna state (for *x* > 0.1) as compared to the scenario when the value of *y* is held constant at 0.1, as clearly evident from Fig. 4(b). Thus, the ability of IS to consider the local dynamics around an attractor, thus taking the aspect of resistance into account, further escalates its potential as a quantifier of multistability.

**Figure 5:**
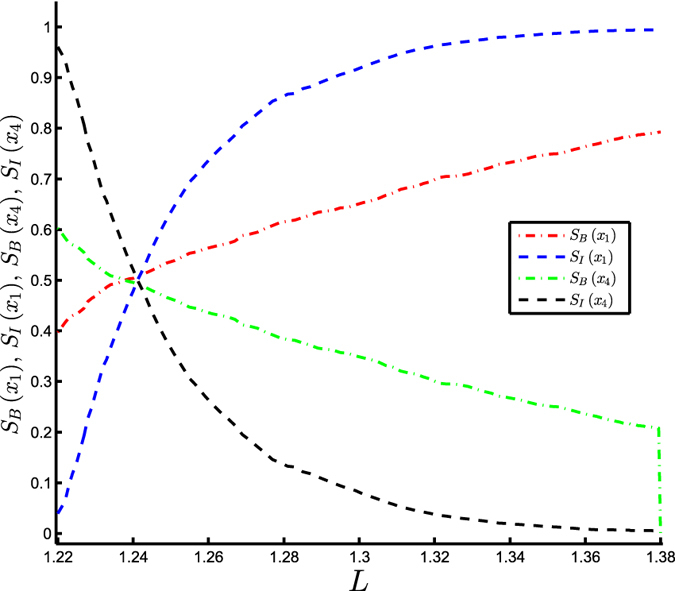
Stability of the Daisyworld model (equation (7)). Integral stability *S*_*I*_(*x*_1_) (blue) [*S*_*I*_(*x*_4_) (black)] and basin stability *S*_*B*_(*x*_1_) (red) [*S*_*B*_(*x*_4_) (green)] of the abiotic solution *x*_1_[bioticsolution *x*_4_] vs. Luminosity (*L*).

Thus far, we have highlighted the capacity of IS in effectively capturing the three crucial aspects of *L*, *R* and *Pr*. Now, we want to further highlight one of the most important benefits of IS which lies in its ability of arresting a gradual loss of the stability of a system (as opposed to an abrupt one captured by BS) when approaching a tipping point. For example, in the Amazonian rainforest, the forest state ceases to exist for *A* > 0.8 as evident from Fig. 3 where the entire state space is occupied by the savanna state. This transition is indicated by the BS of the forest state abruptly dropping to 0 with no convincing early-warning signal. On the contrary, IS demonstrates a gradual and continuous decline in the stability of the forest state. Moreover, it provides a precursor/early-warning signal by capturing a reduced value of the stability of the forest state much prior to the value of *A* = 0.8. This feature of IS is also demonstrated below for the case of the Daisyworld model (Fig. 5).

### Daisyworld

The Daisyworld model relates to interactions between the climate and the biosphere of a hypothetical world orbiting around a star that becomes brighter with time^20^,^21^. The biosphere inhabiting the planet comprises only white and black daisies differing characteristically in their albedo (i.e., the proportion of the incident light or energy they reflect back). The surface covered with white daisies has a higher albedo and reflects a larger proportion of the incoming radiation from the star as compared to that reflected by the bare ground or the surface covered with black daisies. Effectively, the fractional coverages of the planetary area by white and black daisies, denoted by *x*_*w*_ and *x*_*b*_, respectively, determine the overall planetary albedo which subsequently modulates the surface temperature of the planet. Let *p* be the fraction of the fertile ground in the Daisyworld such that *x*_*g*_ ≡ *p* − *x*_*w*_ − *x*_*b*_ constitutes the proportion of uninhabited fertile ground. In the following, we set *p* = 1 for convenience. The dynamical equations comprising the original Daisyworld model^20^, describing the growth of the daisies read


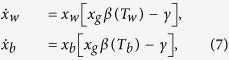


where *γ* is a constant death rate. The birth rate of the white (black) daises denoted by *β*(*T*_*w*_) (*β*(*T*_*b*_)) depends on the local temperature *T*_*w*_ (*T*_*b*_) experienced by the respective daisy type as,


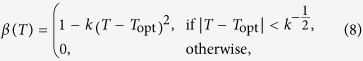


where *k* expresses the sensitivity of the birth rate to the local temperature and *T*_opt_ is the optimal temperature for daisy growth. Denoting the albedo of white and black daisies and that of bare ground by *a*_*w*_, *a*_*b*_ and *a*_*g*_, respectively, we obtain the mean planetary albedo as,


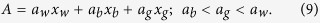


The local temperatures, *T*_*w*_ and *T*_*b*_ depend on the effective planetary temperature *T* as,


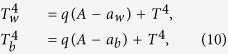


where the parameter *q* is introduced as the heat transfer coefficient. Finally, the mean planetary albedo (*A*), the average energy incident on the surface of the planet radiating from the star (*S*_0_) and the luminosity (*L*) determine the effective planetary temperature (*T*) via the global energy balance equation,


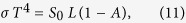


where *σ* is the Stefan-Boltzmann constant. For the fixed parameter values of *σ* = 5.67 × 10^−8^ *Wm*^−2^*K*^−4^, *S*_0_ = 917 *Wm*^−2^, *γ* = 0.3 *s*^−1^, *T*_opt_ = 295.5 *K*, *k* = 3.265 × 10^−3^ *K*^−2^*s*^−1^, *a*_*w*_ = 0.75, *a*_*b*_ = 0.25, *a*_*g*_ = 0.5, *q* = 2.06 × 10^9^ *K*^4^, and *L* ∈ (1.22, 1.38), the Daisyworld model of equation (7) exhibits multistability by admitting four equilibrium points:

- a stable abiotic solution implying complete in-existence of daisies, 
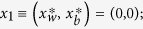
- two saddles with white daisy-only solutions, 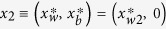 and 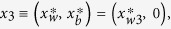 where 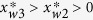;
- a stable biotic solution implying co-existence of daisies, 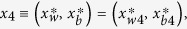 where 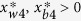.

The measures of IS, *S*_*I*_(*x*_1_) (*S*_*I*_(*x*_4_)), and BS, *S*_*B*_(*x*_1_) (*S*_*B*_(*x*_4_)), of the abiotic solution *x*_1_(bioticsolution *x*_4_) are depicted in Fig. 5. Note that the system switches the state having greater stability at some intermediate value of *L* ∈ (1.22, 1.38). This transition is clearly detected by both measures. The biotic solution *x*_4_ with coexisting daisy populations ceases to exist when the luminosity exceeds a value of 1.38, where the white daisy-only solution *x*_3_ becomes stable. As a result, *S*_*B*_(*x*_4_) abruptly plummets to 0 when *L* > 1.38, indicating a discontinuous loss in the stability of this solution. However, the IS measure, *S*_*I*_(*x*_4_) (*S*_*I*_(*x*_1_)), detects the gradual loss (gain) in the stability of the biotic (abiotic) solution depicted by a very smooth transition to ≈0(1) much prior to the abrupt transition suggested by BS. We have identified this feature of IS earlier in the case of the Amazonian rainforest model (Fig. 3) as well. Thus, the capacity of IS in pronouncing a gradual loss/gain in the stability of an equilibrium state (as well as much prior to that captured by BS) can be a potential precursor/early-warning signal to such transitions in dynamical systems.

## Discussion

The present work was motivated by the pervasiveness of multistability in dynamical systems and the associated need for suitable quantifiers of the stability of multiple attractors of such systems. We redefine the three crucial aspects of ecological resilience (*L*, *R* and *Pr*) for generic dissipative dynamical systems (i.e., systems with attractors) and utilize it as a foundation for characterizing multistability. Subsequently, identifying the inability of the state of the art measures of multistability in collectively capturing *L*, *R* and *Pr*, we propose the novel measure of IS in equation (3).

IS has demonstrated its capacity of capturing a gradual loss of the stability of a system when approaching a transition point as opposed to BS, which exhibits a rather abrupt transition in the same setting. Thus, IS provides a potential early-warning signal prior to such qualitative changes in the dynamics of a complex system making it a prospective tool for assessing such situations arising in many dynamical systems from various fields of application.

In the examples presented above, IS has been applied to low-dimensional systems. We foresee that future efforts could be directed towards probing multistability in high-dimensional systems as well. As an initial exercise in this direction, we apply IS to a multistable high-dimensional system (see Supplementary Information) where we demonstrate that IS appropriately quantifies the associated multistability. Thus, the scope of IS is not associated with the dimensionality of the complex system under assessment.

Immediate potential applications of IS constitute its extension to assessing multistability in networked dynamical systems. For example, the development of a statistical measure similar to the idea of single-node BS proposed by Menck *et al.*^22^ is one feasible direction. We emphasize that the calculation of IS (as well as BS) is computationally expensive (see the Methods section for a discussion on computational complexity associated with the evaluation of IS). Subsequently, future studies should address the development of efficient computational strategies for calculating IS. In this context, any analytical framework supporting the estimation of IS from the dynamical equations of motion will be highly rewarding as well as may significantly reduce the associated computational costs.

The present architecture of IS is applicable to deterministic stationary dissipative dynamical systems. Although being faced with additional practical challenges, the development of a methodology for estimation of IS from time series data sets seems extremely promising. Corresponding in-depth investigations should be the subject of future studies. In this direction, the approach of Tanaka *et al.*^23^ in identifying the separatrices in state space and the basins of attraction from time series data sets may be useful. Further, we identify the work of Abarbanel *et al.*^24^ as an important cornerstone in calculating the local Lyapunov exponents from the observed data sets.

## Methods

### Local Lyapunov Exponents

In the following, we present details on calculating the local Lyapunov exponents as a prerequisite for computing the measure of IS in equation (3). Recall that the resistance at a particular point in state space was defined as the instantaneous rate at which the system converges to the unperturbed trajectory (starting from the state space point in question) following a perturbation. Further, the rate of convergence of nearby trajectories (on account of perturbations) to the trajectory starting at the state space point in question is associated with the negative of the local Lyapunov exponents evaluated at the respective point. Below, we outline the procedure for computing the local Lyapunov exponents at a given point in state space of a continuous dynamical system. However, the procedure remains essentially the same for a discrete dynamical system.

Consider an *N*-dimensional flow (represented by the state vector **x**(*t*) = (*x*_1_, *x*_2_, …, *x*_*N*_)^T^) described by the following equations of motion:


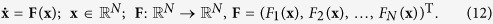


Consider a trajectory starting at **x**, with an infinitesimal perturbation *δ***x**. The perturbation is then transported by the flow **F** along the (perturbed) trajectory starting at **x** + *δ***x**. We study the deformation of the infinitesimal neighbourhood using the flow linearised around **x**, following from the variational equations obtained by Taylor expansion of equation (12) to first order,


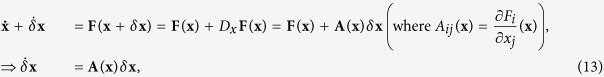


where **A**(**x**) is the stability matrix, describing the instantaneous rate of shearing of the neighbourhood of **x** by the flow. Since resistance measures the instantaneous rate at which the perturbation grows (or decays), we calculate the growth of the perturbation for an infinitesimal change in time *dt*,


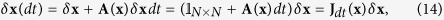


where **J**_*dt*_ = 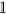
_*N*×*N*_ + **A**(**x**)*dt* is the instantaneous Jacobian matrix. It describes the deformation of an infinitesimal neighbourhood in time *dt* of the trajectory starting at **x**. For a map, the instantaneous Jacobian matrix simply consists of the partial derivatives of the map with respect to the state variables, evaluated at the respective point in state space.

The square roots of the eigenvalues of the right (left) Cauchy-Green strain tensor, 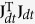 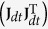, called the ‘principal stretches’ and denoted by *σ*_*j*_(**x**), *j* = 1, 2, …, *N*, measure the instantaneous stretching of the neighbourhood of the trajectory at **x**. The local Lyapunov exponents measuring the rate of stretching are given by^25^,


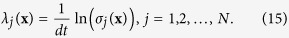


### Computational Complexity

The calculation of IS entails the numerical estimation of the basin structure, computation of local Lyapunov exponents and precariousness of all attractors of the system (in order to obtain the normalization constant of equation (3)). For an *N*-dimensional system, we estimate the basin of each attractor using a numerical Monte-Carlo procedure by drawing *I*^*N*^ random initial states, simulating the associated trajectories, and assigning each initial state to the respective basin of the attractor it finally approaches. We recommend using a value of *I* = 100 as this yields a standard error proportional to 
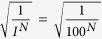
 which is typically very small^9^. The calculation of local Lyapunov exponents and precariousness of all attractors using the methods previously outlined is computationally effortless and only takes a few minutes on a standard laptop computer. The estimation of the basin structure is the bottleneck in terms of computational time required for calculating IS. For example, for the low-dimensional systems treated here, the estimation of basin structure for a given set of parameter values takes approximately 30 minutes of calculation using sequential programming in MATLAB. As a first illustration of the computational feasibility, we also apply IS to a multistable high-dimensional system (see Supplementary Information) where we demonstrate that IS appropriately quantifies the associated multistability and is not directly restricted by the dimensionality of the system. However, the computational time for the estimation of the basin structure and, hence, IS increases exponentially with the dimensionality of the system. In order to overcome these practical limitations, future studies should employ more efficient computational strategies for calculating IS.

## Additional Information

**How to cite this article**: Mitra, C., Kurths, J. & Donner, R. V. An integrative quantifier of multistability in complex systems based on ecological resilience *Sci. Rep.* **5**, 16196; doi: 10.1038/srep16196 (2015).

## Supplementary Material

Supplementary Information

## Figures and Tables

**Figure 1:**
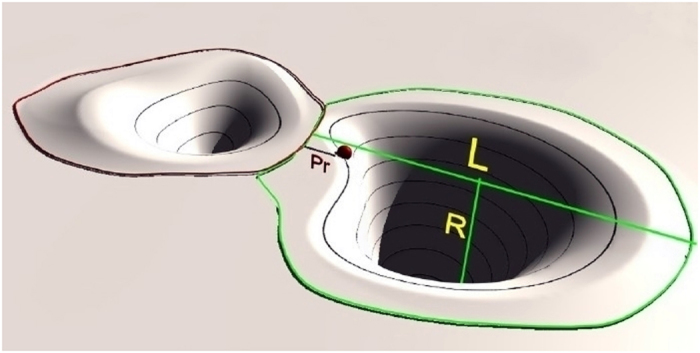
Crucial aspects of (ecological) resilience. Stability landscape of a bistable dynamical system consisting of two state variables (forming the two axes of the stability landscape while resistance, i.e., the depth of the basin of attraction constituting the vertical axis) showing the current state of the system (red dot) and three aspects of resilience: *L* = Latitude, *R* = Resistance, *Pr* = Precariousness as defined by Walker *et al.*^13^ (Adapted from Leuteritz *et al.*^26^). A deep basin of attraction (*R*) or, more precisely, a steeper slope at a point indicates that larger forces or perturbations are required to change the state of the system around that point. The bottoms of each of the two basins (either of which the system equilibrates to) are heuristic representations of the attractors of the bistable system while the red and green lines mark the basin boundaries. The smaller (larger) spacing between successive black contour lines in the neighbourhood of a particular point implies a steeper (shallower) slope of *R* or equivalently, greater (lesser) management efforts required to move the system around that point.
