# Supplementary material for: An integrative quantifier of multistability in complex systems based on ecological resilience
Source: Sci Rep. 2015 Nov 5;5:16196. doi: 10.1038/srep16196 (PMC4633666; doi:10.1038/srep16196)
Supplement: Supplementary Information [file srep16196-s1.pdf]

Supplementary Information: An integrative quantifier of multistability in complex systems based on ecological resilience

Chiranjit Mitra[1, 2, *], Jürgen Kurths[1, 2, 3, 4], and Reik V. Donner[1, *]

[1]Potsdam Institute for Climate Impact Research, Transdisciplinary Concepts & Methods - Research Domain 4, Potsdam, 14412, Germany
[2]Humboldt University of Berlin, Department of Physics, Berlin, 12489, Germany
[3]University of Aberdeen, Institute for Complex Systems and Mathematical Biology, Aberdeen, AB24 3UE, United Kingdom
[4]Nizhny Novgorod State University, Department of Control Theory, Nizhny Novgorod, 606950, Russia
[*]chiranjit.mitra@pik-potsdam.de, reik.donner@pik-potsdam.de

ABSTRACT

In the main paper, we applied integral stability to several exemplary but low-dimensional systems in order to demonstrate its potential as an effective quantifier of multistability. In this Supplementary Information, we illustrate that the scope of this measure is not associated with the dimensionality of the system. For this purpose, we demonstrate its application to a multistable high-dimensional dynamical system.

Further Example: High-dimensional dynamics

We consider a chain of N one-variable bistable cubic Nagumo systems[1] where the evolution of each isolated unit is given by

$$\dot{x} = x(x-\eta)(1-x), \text{ with } 0 < \eta < 1. \tag{1}$$

We assume a nearest-neighbour coupling between the systems with a coupling strength denoted by α (>0) such that the dynamical equations read

$$\dot{x}_i = x_i(x_i-\eta)(1-x_i) + \alpha\,(x_{i+1} + x_{i-1} - 2x_i)\,,\ i = 1, 2, \dots, N. \tag{2}$$

The coupled system of equations (2) with (at least) two stable equilibria at

$$\begin{aligned} X_1^* &: x_i^* = 0,\ \forall\, i = 1, 2, \dots, N, \\ X_2^* &: x_i^* = 1,\ \forall\, i = 1, 2, \dots, N, \end{aligned} \tag{3}$$

has been investigated in greater detail by Mackay et al.[1] We refer the reader to this original study for further details on the dynamics of the model.

Here, we analyse the model in equation (2) for a sufficiently large number of $N = 50$ units for it to qualify as a high-dimensional system. We utilize integral stability to quantify the stability of the bistable stationary states of the network (equation 3). The measures of integral stability, $S_I(X_1^*)$ $(S_I(X_2^*))$ and basin stability, $S_B(X_1^*)$ $(S_B(X_2^*))$ of the equilibrium point X_1^* (X_2^*) vs. η at a value of $\alpha = 0.5$ are depicted in Supplementary Fig. S1. Just like the Daisyworld model addressed in the main text, the system in equation (2) switches the state having greater stability at some intermediate value of $\eta \in (0, 1)$. This transition is clearly detected by both measures. As clearly evident from Supplementary Fig. S1, the measure of integral stability closely follows the measure of basin stability. Thus, we conclude that integral stability is equally well applicable to a high-dimensional system (just like basin stability) and that it is not prone to the curse of being dimensionally restrictive.

References

1. MacKay, R. & Sepulchre, J.-A. Multistability in networks of weakly coupled bistable units. *Physica D: Nonlinear Phenomena* **82**, 243–254 (1995).

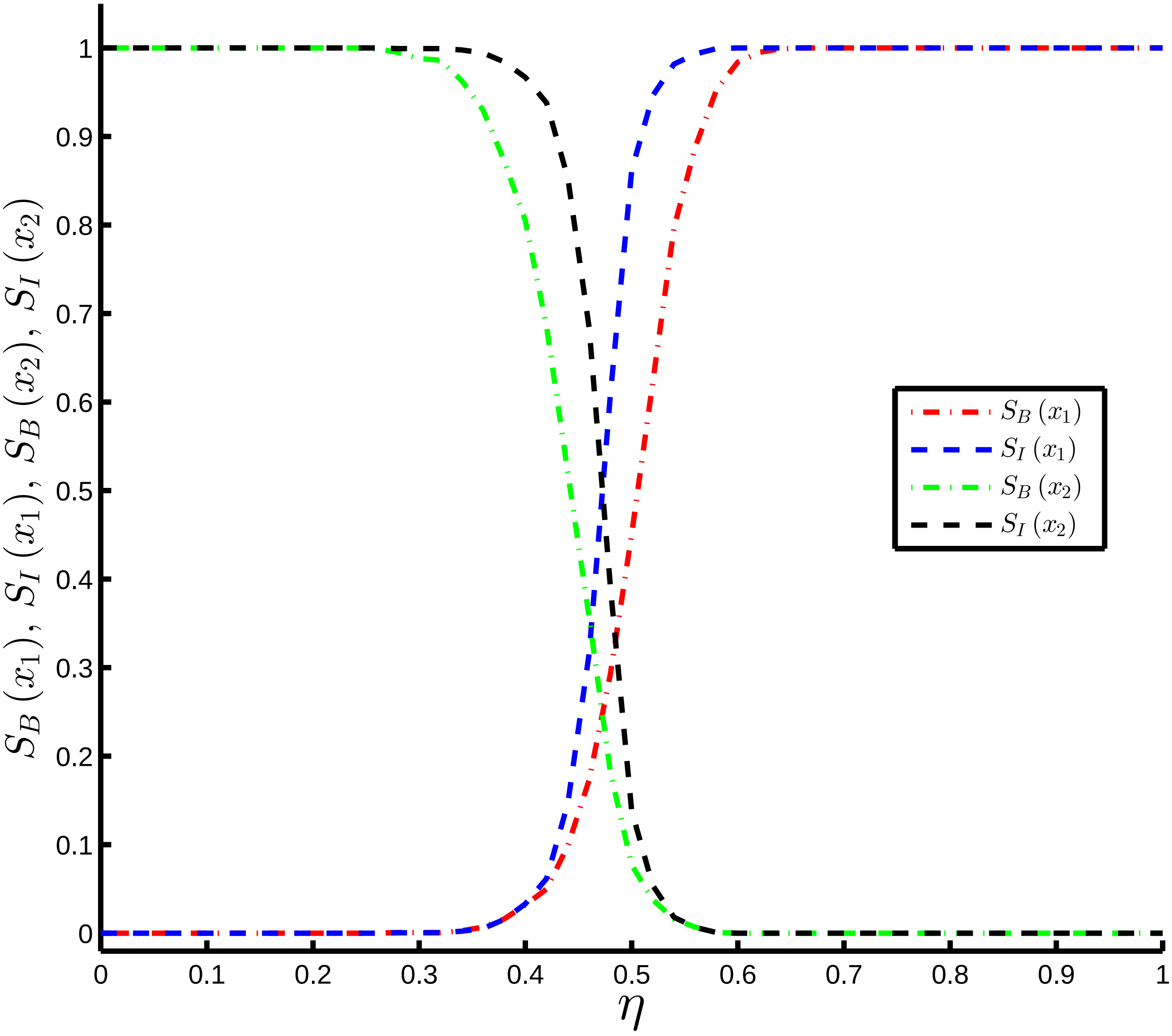

Figure S1. Stability of the cubic Nagumo model network (equations (2)). Integral stability $S_I(X_1^*)$ (blue) [$S_I(X_2^*)$ (black)] and basin stability $S_B(X_1^*)$ (red) [$S_B(X_2^*)$ (green)] of the equilibrium point X_1^* [X_2^*] vs. η, at $\alpha = 0.5$.
